# High-Quality Draft Genomes of Two *Vibrio parahaemolyticus* Strains Aid in Understanding Acute Hepatopancreatic Necrosis Disease of Cultured Shrimps in Mexico

**DOI:** 10.1128/genomeA.00800-14

**Published:** 2014-08-14

**Authors:** Silvia Gomez-Jimenez, Lorena Noriega-Orozco, Rogerio R. Sotelo-Mundo, Vito A. Cantu-Robles, Ana G. Cobian-Guemes, Rosario G. Cota-Verdugo, Luis A. Gamez-Alejo, Luis del Pozo-Yauner, Eduardo Guevara-Hernandez, Karina D. Garcia-Orozco, Alonso A. Lopez-Zavala, Adrián Ochoa-Leyva

**Affiliations:** a Centro de Investigación en Alimentación y Desarrollo AC (CIAD), Hermosillo, Sonora, México; b Centro de Investigación en Alimentación y Desarrollo AC (CIAD), Guaymas, Sonora, México; c Instituto Nacional de Medicina Genómica (INMEGEN), México, D.F., México; d Unidad de Genómica de Poblaciones Aplicada la Salud, Facultad de Química, UNAM, Instituto Nacional de Medicina Genómica (INMEGEN), México, D.F., México

## Abstract

The high-quality draft genomes of two *Vibrio parahaemolyticus* strains, one that causes the acute hepatopancreatic necrosis disease (AHPND) in cultured shrimps (FIM-S1708^+^), and another that does not (FIM-S1392^−^) are reported. A chromosome-scale assembly for the FIM-S1392^−^ genome is reported here. The analysis of the two genomes gives some clues regarding the genomic differences between the strains.

## GENOME ANNOUNCEMENT

Great effort has been undertaken to understand the molecular basis of the devastating bacterial acute hepatopancreatic necrosis disease (AHPND), also called early mortality syndrome (EMS), which is affecting the global culture of shrimp. Taking a different approach, we have isolated a bacterial collection from shrimp culture pond sediments. Two strains were selected for genome analysis, considering their ability to cause or not cause AHPND/EMS based on shrimp bioassays (1) and for being positive or not to the PCR detection method (2) and positive to AP2 primers (3). Genomic DNA of the disease-causing strain (FIM-S1708^+^) and the innocuous strain (FIM-S1392^−^) of *Vibrio parahaemolyticus* were extracted with a kit from Zymo. Each DNA strain was independently sequenced by AXEQ (Korea) using the Illumina HiSeq 2000 and by the Instituto Nacional de Medicina Genomica (INMEGEN) (México) using the Illumina GaIIx. The AXEQ libraries, containing a 280-bp insert, were sequenced with the paired-end protocol of 100-bp reads, and the INMEGEN libraries, containing a 300-bp insert, were sequenced with the paired-end protocol of 72-bp reads. The total filtered paired-end reads generated from the FIM-S1392^−^ and FIM-S1708^+^ libraries were 1,411 Mb and 1,319 Mb, respectively. The genome of FIMS1392^−^ was assembled into 14 scaffolds (*N*_50_, 1,931,617 bp) and 65 contigs >1,000 bp (*N*_50_, 215,659 bp), and that of FIM-S1708^+^ was assembled into 79 contigs >1,000 bp (*N*_50_, 174,266 bp) using Velvet. The largest contigs are 2,406,161 bp for FIM-S1392^−^ and 629,794 bp for FIM-S1708^+^. The total lengths of the FIM-S1392^−^ and FIM-S1708^+^ genomes are 5,174,919 bp (22× coverage) and 5,246,988 bp (25× coverage), respectively.

After assembly, the contigs from both strains were mapped to two chromosomes of *V. parahaemolyticus* RIMD2210633 using CONTIGuator. The analysis of the FIM-S1392^−^ genome showed that scaffold13 (containing 2,406,161 bp) covers 73% of the RIMD2210633 chromosome 1, and scaffold11 (containing 1,931,617 bp) covers 100% of the RIMD2210633 chromosome 2. A comparison of the FIM-S1708^+^ genome showed that contig22 (containing 629,794 bp) covers 19% of the RIMD2210633 chromosome 1, and contig62 (containing 406,842 bp) covers 22% of the RIMD2210633 chromosome 2. The two strains lack the 140-kbp region in chromosome 2 (positions 1387705 to 1467746) that contains genes encoding a type III secretion system. This region is also deleted in other *V. parahaemolyticus* isolates from AHPND-diseased shrimps in Thailand (4, 5). The comparative genomic analysis of the FIM-S1708^+^ and FIM-S1392^−^ genomes against RIMD2210633 also revealed the presence of genes coding for the prophage f237 in chromosome 1 of FIM-S1708^+^ and showed that this genomic region is absent in FIM-S1392^−^. Additionally, FIM-S1392^−^ lacks a genomic region in chromosome 2 that contains several putative phage-related proteins, while this region is present in the FIM-S1708^+^ and RIMD2210633 genomes. Further studies will confirm the genomic changes that are involved in the pathogenicity of the FIM-S1708^+^ strain. Most of the observed differences between the genomes correspond to plasmid sequences and to several clusters mainly featuring phage-related genes.

### Nucleotide sequence accession numbers.

This whole-genome shotgun project has been deposited at DDBJ/EMBL/GenBank under the accession numbers JPLV00000000 and JPLU00000000. The versions described in this paper are JPLV01000000 and JPLU01000000.

## References

[1] Tran L Nunan L Redman RM Mohney LL Pantoja CR Fitzsimmons K Lightner DV 2013 Determination of the infectious nature of the agent of acute hepatopancreatic necrosis syndrome affecting penaeid shrimp. Dis. Aquat. Org. 105:45–55. 10.3354/dao02621 23836769

[2] Nunan L Lightner D Pantoja C Gomez-Jimenez S 2014 Detection of acute hepatopancreatic necrosis disease (AHPND) in Mexico. Dis. Aquat. Organ., in press. 10.3354/dao02776 25144120

[3] Joshi B Srisala J Truong VH Chen IT Nuangsaeng B Suthienkul O Lo CF Flegel TW Sritunyalucksana K Thitamadee S 2014 Variation in *Vibrio parahaemolyticus* isolates from a single Thai shrimp farm experiencing an outbreak of acute hepatopancreatic necrosis disease (AHPND). Aquaculture 428–429:297–302, 10.1016/j.aquaculture.2014.03.030

[4] Kondo H Tinwongger S Proespraiwong P Mavichak R Unajak S Nozaki R Hirono I 2014 Draft genome sequences of six strains of *Vibrio parahaemolyticus* isolated from early mortality syndrome/acute hepatopancreatic necrosis disease shrimp in Thailand. Genome Announc. 2(2):e00221-14. 10.1128/genomeA.00221-14 24723705 PMC3983294

[5] Gomez-Gil B Soto-Rodríguez S Lozano R Betancourt-Lozano M 2014 Draft genome sequence of *Vibrio parahaemolyticus* strain M0605, which causes severe mortalities of shrimps in Mexico. Genome Announc. 2(2):e00055-14. 10.1128/genomeA.00055-14 24604636 PMC3945492

